# Climate Change Helps Polar Invasives Establish and Flourish: Evidence from Long-Term Monitoring of the Blowfly *Calliphora vicina*

**DOI:** 10.3390/biology12010111

**Published:** 2023-01-10

**Authors:** Ella Z. Daly, Hannah Sørine Gerlich, Yves Frenot, Toke T. Høye, Martin Holmstrup, David Renault

**Affiliations:** 1 UMR CNRS 6553 ECOBIO (Ecosystèmes, Biodiversité, Evolution), Université Rennes, Avenue du Général Leclerc, 35042 Rennes, France; 2 Department of Ecoscience and Arctic Research Centre, Aarhus University, C.F. Møllers Allé 4-8, DK-8000 Aarhus, Denmark

**Keywords:** biological invasion, sub-Antarctic, Calliphoridae, climate change, phenology, degree day, thermal requirements, insect development, ectotherm

## Abstract

**Simple Summary:**

Sub-Antarctic islands, such as the Kerguelen Archipelago, are home to a unique collection of species because of their isolation. However, their isolation and low number of species make them more susceptible to invasion. This is particularly important as warming temperatures due to climate change are also making conditions worse for local species, and potentially better for recently introduced species, most of which are from warmer areas. The threats from non-native species in such a sensitive and unique ecosystem makes it important to understand the specific effects of climate change on non-native species to be able to predict their likely local population trajectory. In this study, we explored whether warming temperatures helped a non-native blowfly, *Calliphora vicina*, establish and spread throughout Kerguelen. We found that warming temperatures have made this species better able to survive and reproduce annually, and that it should continue to benefit from warming temperatures. We also discuss the potential impacts of this invasive blowfly on the local ecosystem and recommend further studies to assess its impacts and to identify management options for the species, which is likely to persist locally for the long term.

**Abstract:**

The isolated sub-Antarctic islands are of major ecological interest because of their unique species diversity and long history of limited human disturbance. However, since the presence of Europeans, these islands and their sensitive biota have been under increasing pressure due to human activity and associated biological invasions. In such delicate ecosystems, biological invasions are an exceptional threat that may be further amplified by climate change. We examined the invasion trajectory of the blowfly *Calliphora vicina* (Robineau-Desvoidy 1830). First introduced in the sub-Antarctic Kerguelen Islands in the 1970s, it is thought to have persisted only in sheltered microclimates for several decades. Here, we show that, in recent decades, *C. vicina* has been able to establish itself more widely. We combine experimental thermal developmental data with long-term ecological and meteorological monitoring to address whether warming conditions help explain its current success and dynamics in the eastern Kerguelen Islands. We found that warming temperatures and accumulated degree days could explain the species’ phenological and long-term invasion dynamics, indicating that climate change has likely assisted its establishment. This study represents a unique long-term view of a polar invader and stresses the rapidly increasing susceptibility of cold regions to invasion under climate change.

## 1. Introduction

The transport and release of non-native species has become a global ecological and conservation concern, as invasive organisms are increasingly altering terrestrial and aquatic communities worldwide [1,2]. By facilitating the establishment of non-native species, boosting proliferation, and assisting their geographic expansion through a number of mechanisms, climate change vastly increases the threats incurred by biological invasions [3,4]. Consequently, there is a growing body of evidence reporting that biological invasions can alter ecosystem functioning [5,6], community structure, or interaction networks [7], leading to the homogenization of ecological communities and the loss of native and endemic species [8]. Yet, even if climate change is frequently claimed as being an important driver of establishment success, proliferation, and spread of non-natives, it has often remained difficult to determine the exact contribution of climate change to the invasion processes. Several additional factors, including propagule pressure, introduction efforts, and other forms of global changes, may act as confounding factors [3,9,10]. In particular, nitrogen deposition, and more generally environmental pollution, habitat fragmentation, or changes in land-use, are major factors driving processes, mechanisms, and impacts of biological invasions [11,12]. This makes polar (Arctic, sub-Arctic, Antarctic, and sub-Antarctic regions) and high-elevation regions, which are less subjected to human-induced environmental degradation than other regions of the world, valuable model systems for investigating the impacts of climate change on the success of biological invasions.

Invertebrates are sensitive to warming due to their reliance on external sources of heat. In these organisms, thermal and desiccation tolerances are major factors setting their distribution limits and thermal niches [13]. In Antarctic regions, many of these communities already live at the edge of their climatic limits, thus suggesting that accelerating climate change is likely to increase the threat from and displacement of those species further [14]. In addition, meteorological conditions are expected to become more variable, threatening organisms with narrow thermal and desiccation tolerances, while favouring those exhibiting broader tolerance ranges (climate variability hypothesis) [15], in particular, non-native species. Finally, new thermal niches offered by warming make polar regions more suitable to less stress-adapted non-native species, and the status of some existing non-native species may shift from introduced to invasive [4,13]. Novel conditions due to climate change can also favour non-native species over native species, especially in cold regions. In these ecosystems, cold-adapted native species and non-native species, usually adapted to warmer conditions, may experience contrasting effects from increases in temperature [16,17]. The interaction between climate change and biological invasions can present a significant threat to cold ecosystems.

The seasonal timing of biological events (i.e., phenology) are considered key indicators of organismal responses to climate change [18,19]. As temperatures increase, the phenology of many taxa advances, but little attention has been given to the importance of phenology as an indicator of temporal niches of species in a community. However, as growing seasons expand, vacant temporal niches may become available and allow introduced species to invade native communities. It is increasingly recognized that non-native species are successful invaders as they are often more phenologically sensitive than native species [20]. This allows non-native species to take advantage of windows of invasion opportunity in environments with extended temporal niches where native species are unable to track the shifting climate [21]. The phenological patterns of introduced species and their climatic sensitivity can thus help us understand their ability to establish in a community.

Climate change is particularly significant in polar and alpine regions, and recent warming has been of greater magnitude in these regions than in other parts of the globe [22]. These changes in climatic conditions have already led to poleward range shifts across many taxonomic groups, both on land and in the oceans [19,20]. However, the extent of climate-related range expansions and shifts of non-native species has been less investigated in polar regions than in temperate regions (but see [23]). Although the climate of subantarctic regions is not as cold as other polar regions, the temperatures are constantly low, ranging from about 2 °C in the winter to 8 °C during summer [16], and this can be a challenge for ectothermic species. Increasing local temperatures, which were formerly restrictive for non-native species in this region, could now permit their establishment.

The blowfly *Calliphora vicina* (Robineau-Desvoidy 1830) can be found on all continents, except for Antarctica. Despite its wide distribution, most studies of this species have focused on its development and general biology (e.g., see [24]), owing to the utility of the fly in forensic entomology (e.g., [25,26]). Consequently, knowledge on its invasive characteristics and ecological impacts [27] is sparse. However, other invasive blowflies have known negative effects on natives through competition for carrion resources [28,29]. It is, therefore, likely that *C. vicina* could have negative effects on an ecosystem as sensitive as the Kerguelen Archipelago through competition with native species, the alteration of nutrient dynamics, and the facilitation of future invaders [16,27,30,31]. *Calliphora vicina* was unintentionally introduced to the sub-Antarctic Kerguelen Islands during the 20th century, and long-term monitoring beginning in 1993 has captured its arrival, establishment, and proliferation throughout the archipelago. This presents a unique opportunity to understand the invasion process, shed light on the significance of this non-native species in its introduced range, and gain insights into why this species is so successful globally.

The first known local sighting of *C. vicina* in the Kerguelen Islands occurred in 1978 at the archipelago’s main port and settlement in Port-aux-Français [32]. The individuals were suspected to have arrived from the sub-tropical Réunion Island and, because of harsh environmental conditions at the Kerguelen Islands, it was initially considered unlikely that a self-sustaining population could establish. Ameliorating climatic conditions may have contributed to its eventual establishment approximately 20 years after it was first recorded locally [16]. Prior to the 1990s, seasonal temperatures may simply not have been high enough for *C. vicina* to complete its life cycle; however, this has never been formally examined. The species undergoes a complete metamorphosis with six distinct life stages: egg, three larval instars, pupa, and adult (see [33] for the life cycle diagram). Eggs are deposited during the daytime in carrion, where they hatch and proceed to feed on the tissue in early larval stages. Post-feeding larvae move away from the carrion to pupate in a nearby substrate. This can take place quickly, or after maternally controlled diapause as a third instar larva at temperatures below 15 °C in temperate regions, if the mother is exposed to days of short photoperiod [34]. The species can be univoltine (one generation per year) or multivoltine (multiple generation times per year) depending on environmental conditions [35,36,37].

Since the ecological impacts of the *C. vicina* invasion are unknown and given the high susceptibility of sub-Antarctic island communities to invasion [16,30], and the uniqueness of the ecosystem [32], it is imperative to understand the invasion mechanisms and trajectory of this species to the Kerguelen Islands. In line with this, our first aim was to determine if warming could have facilitated the establishment of the species at the Kerguelen Islands. To that aim, we created a degree day model based on thermal developmental requirements of this strain and used it to assess the suitability of the years pre- and post-establishment for the completion of its life cycle. Our second aim was to analyse long-term, year-round, monitoring data to examine the phenology of *C. vicina* in the Kerguelen Islands and how it relates to temperature. Knowledge on phenological responses of *C. vicina* allows us to better understand how this species can track environmental change, and thus provides insight into the invasion process, trajectory, and potential impacts.

## 2. Materials and Methods

To assess the thermal developmental requirements of individuals of *C. vicina* from Kerguelen, adult flies were trapped around the research station of Port-aux-Français (49°21′00″ S, 70°13′00″ E) in December 2008–January 2009 using traps baited with sheep liver. Breeding flies were then kept in Tupperware^®^ (Nanterre, France) boxes (19 × 19 × 12 cm, l × l × h) with honey, water, and a piece of liver where they could lay eggs. Newly laid eggs were assigned to one of five thermal conditions for incubation (4, 8, 12, 16, and 20 °C) and a control treatment under ambient outdoor temperatures (ambient conditions, AC). For each temperature condition, 3–4 groups of 20 eggs were transferred using a small brush to rearing chambers, where they were placed on raw liver in small boxes containing sand and a piece of damp cotton. Daily observations of the number of individuals in each growth stage were recorded from this point until the emergence of adult flies, except for the 4 °C incubation group, where eggs hatched too late to be included in the experiment (after 31 days). The experiment was repeated at least three times for each thermal condition.

Degree day models use accumulated degree days, a unit of heat/time (usually one degree over the minimum developmental temperature of an organism held for 24 h), to predict when a population will reach a particular life stage [38]. We calculated the number of degree days required for *C. vicina* to reach each developmental stage from the thermal development experiments. Using this data, we created a degree day model to assess whether the degree days accumulated during the austral summer were sufficient for this species to complete its life cycle during the years pre- and post-establishment at the Kerguelen Islands. Regional studies found that *C. vicina* can develop at temperatures as low as 1 or 2 °C see [39] for a brief review, depending on regional adaptations, and we selected 2 °C as a conservative minimum developmental temperature. Evidence from other regions suggests an upper developmental threshold of 28 °C [26], which we also applied, although individuals are unlikely to experience such high temperatures on the Kerguelen Islands.

We parameterized our degree day model with the number of degree days required for development, and lower and upper developmental thresholds. We used the 200th day of each civil year as the start of the accumulation of degree days, which marks the beginning of the austral winter prior to the breeding season. A meteorological station located at the Port-aux-Français research station measured air temperature throughout the entire study period (at 2 m height before 2006 and at 10 cm height from 2006 and onwards). Comparisons between soil temperature (depth 5 cm) and air temperature (measured at 2 m height) revealed minimal differences between the two measurements at the time of the study (project 136-SUBANTECO, French Polar Institute), and thus, we did not apply an offset for soil temperature. We used this degree day model to calculate how many days into the breeding season the flies would have accumulated enough degree days to complete their life cycle, i.e., to determine which years the flies should be able to persist and breed during the austral summer. We also performed a *t*-test of the number of degree days accumulated in the austral summer of years pre- and post-introduction to see if there was a significant difference.

Long-term monitoring of adult *C. vicina* phenology at Port-aux-Français took place from 1993 to 2020 using a baited trap. Adult *C. vicina* lay their eggs in carcasses of native seabirds and introduced vertebrate species (including rabbits (*Oryctolagus* spp., Lilljeborg 1873), mice (*Mus* spp., Clerck 1757), sheep and mouflons (*Ovis* spp., Linnaeus 1758), and reindeer (*Rangifer tarandus*, Linnaeus 1758)). The larvae grow in the carrion until the third instar larva migrates into the soil beneath the carrion to pupate [40]. Thus, the baited trap contained a box with sheep liver (5 cm^3^) to attract adult *C. vicina* for oviposition. The bait was changed approximately every three months when it was deemed too decayed. The sampling frequency in the permanent trap changed across years. In the years 1993–1995, the trap was emptied approximately every second week from January to June. In the years 1996–1999, the trap was emptied every second week all year round. In the years 2000–2020, the trap was emptied every five days from November to April, and every seven to ten days from May to October. After collection, the samples were sorted in the laboratory at the Port-aux-Français research station, and the numbers of adult flies were counted. In the present work, adult counting was used to examine the local phenology of the species. The temporal trend in annual average air temperature recorded was analysed using generalised additive models (GAMs), which allowed us to capture complex patterns in temperature across the seven-decade time series data [41].

Individuals of *C. vicina* were also monitored during general invertebrate surveys on the Kerguelen Islands during the years 2004–2021. Different observation and collection methods were used during the general invertebrate surveys, including pitfall traps (Port-aux-Français, Isthme-Bas, Guillou Island, Verte Island, Cochons Island), yellow traps (Port-aux-Français), and soil samples and visual observations (over the whole of the Kerguelen Islands). For each observation, GPS coordinates (WGS 84) and dates were noted and used to describe the spatial and temporal distribution trends of *C. vicina* at the Kerguelen Islands. As the geographical location of observations of specimens of *C. vicina* were not consistent across the years, the data did not allow us to show repeated occurrence points on a specific location. Therefore, in the few cases where repeated annual observations of *C. vicina* were made on the same location, the occurrence points on the map represented the time when the first observation was made. To map the temporal changes in the geographical distribution of *C. vicina*, we used QGIS version 3.22.5 [42].

**Figure 1:**
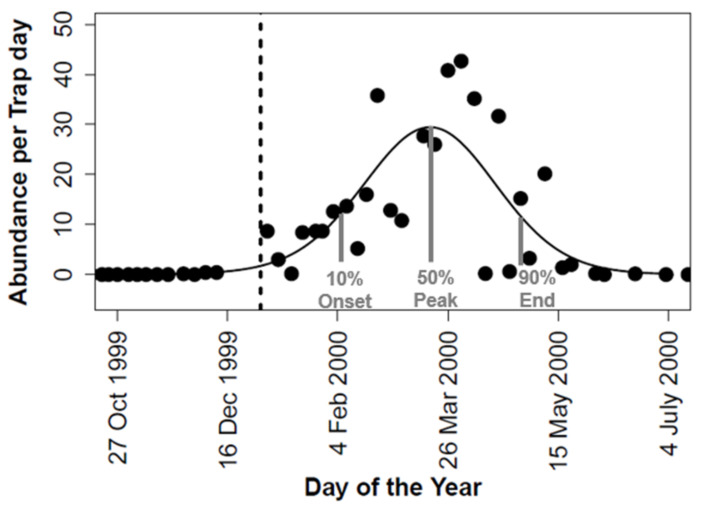
Example of fitting of a phenological curve using generalised additive models for abundance data of adults of the blowfly *Calliphora vicina* to estimate onset, peak, and end of emergence. This example is based on data for abundance per trap day for *C. vicina* collected in the years 1999–2000. The dashed vertical line indicates the start of the calendar year.

To assess the interannual variation in the timing of adult activity in *C. vicina*, we generated phenological curves of capture rates for each breeding year using GAMs assuming a poisson distribution (Figure 1) using the GAM implementation in the mgcv package in R version 4.2.1 [43]. We used partial smoothing functions in GAMs to model the seasonal activity because the exact shape was unknown. The annual onset, peak, and end of the activity season were then calculated as the date at which 10%, 50%, and 90% of the accumulated number of adult *C. vicina* (area under the curve) were trapped, respectively (Figure 1). We restricted our analysis to years where individuals were present in at least two weeks, and where a realistic seasonality curve could be generated. The years 1994, 2017, and 2020 were thus excluded from the analysis due to missing information on dates with zero captures or where the seasonality curves were clearly bimodal (Figure S1). In addition, the trap was closed in the years 2003 and 2015, and, therefore, no data was available for these years.

## 3. Results

### 3.1. Thermal Requirements of Calliphora vicina Individuals from the Kerguelen Islands

The developmental temperature can affect different life cycle parameters, and a higher temperature generally accelerates development, in turn, reducing the time needed to reach each developmental stage. In our experiment on the influence of temperature on developmental time, we also observed that early developmental stages (hatching and the first two larval stages) were generally shorter than later stages (Table 1). Using this data, we calculated the number of degree days needed to reach each stage of development (Table 1) and we calculated the number of accumulated degree days to complete the fly’s life cycle (mean required degree days over the tested thermal gradient: 439 degree days).

### 3.2. Thermal Trends on the Kerguelen Islands over Previous Seven Decades

**Figure 2:**
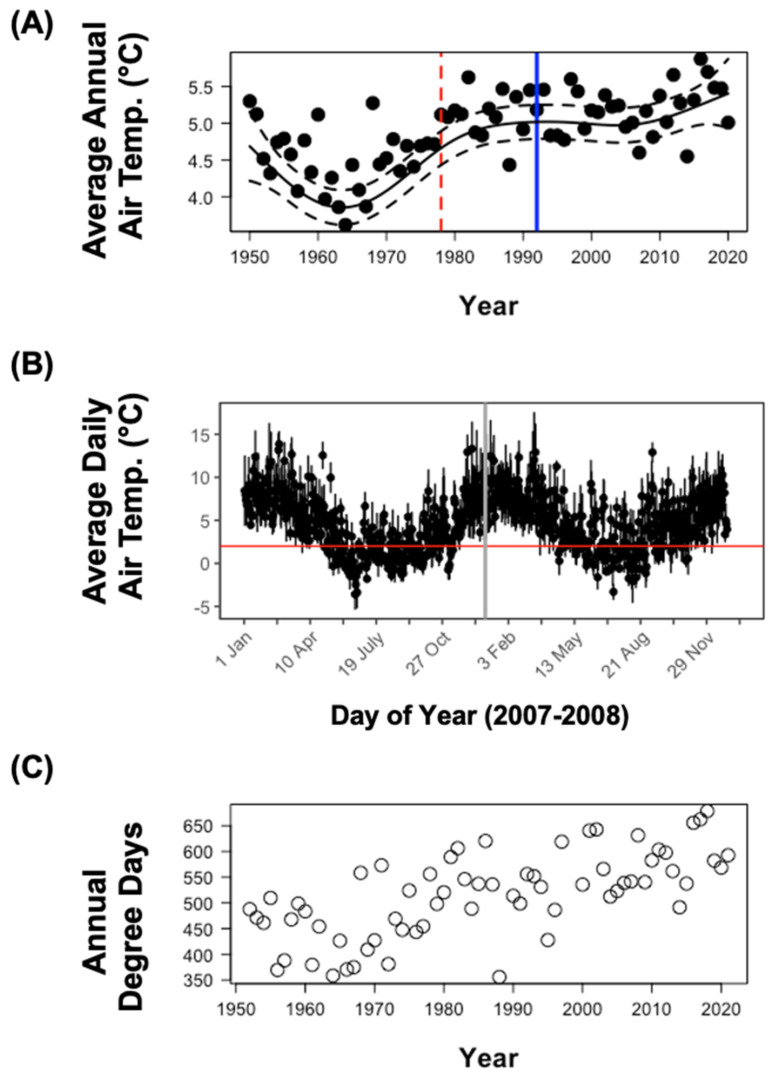
Temperature trends at the Kerguelen Islands and comparison of accumulated degree days with estimated thermal requirements of *C. vicina*. (**A**) Annual average air temperature from 1950–2020 (the vertical red dashed line indicates when *C. vicina* was first observed at the archipelago and the blue vertical line indicates when the monitoring of *C. vicina* was initiated). (**B**) Daily temperature fluctuations across two example years (2007–2008) with vertical grey line indicating the start of the calendar year and the horizontal red line indicating the assumed minimum developmental temperature (2 °C). (**C**) Degree days accumulated by the end of the breeding season calculated from 200th day of year (austral mid-winter when adult flies are inactive) to end of February of following year, based on daily temperatures. Temperature data from Météo France.

Air temperatures generally increased at the Kerguelen Islands from the 1950s onwards, with a marked difference before and after the 1970s (Figure 2A), and a potential plateau from this period until 2021. The warmest year on record was 2016, when an average annual air temperature of 5.9 °C was recorded. This is a difference of 2.2 °C compared to the coolest average annual air temperature (3.6 °C) recorded in 1964. The GAM model explained 53.8% of the deviance and the smoothing spline of year was significant, indicating a non-linear increase in temperature across years. Average daily air temperatures exhibited more variability than average annual air temperatures, as demonstrated in the example plot for 2007–2008 (Figure 2B) and were frequently above the assumed minimum developmental threshold for *C. vicina* (2 °C) in more recent years. The daily temperature peaks could contribute significantly to the rapid accumulation of degree days for *C. vicina*.

With the increasing temperatures, we also observed an increase in the degree days accumulated during the austral summer (Figure 2C). Using a *t*-test, we found that the degree days accumulated by the end of the austral summer had significantly increased in years following 1978 (t_68_ = 4.67, *p* < 0.0001), when this species was first observed locally (Figure 2C). The number of degree days accumulated in the austral summer has almost always been above 450 degree days during the last three decades (Figure 2C), indicating that this population should have been able to complete its life cycle in most years.

### 3.3. Long-Term Spatial and Temporal Monitoring of Calliphora vicina in the Kerguelen Islands

**Figure 3:**
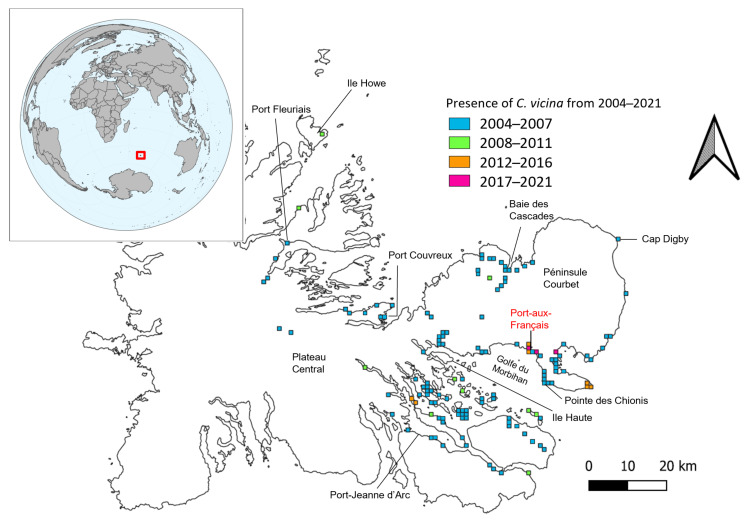
Changes in the distribution of *Calliphora vicina* on the Kerguelen Islands in the time range 2004–2021. The occurrence points report the period of first observations of the presence of *C. vicina*. Note that in most localities, the species was observed repeatedly in the periods following its initial observation. Distribution map generated in QGIS version 3.22.5 with observations plotted on a one-kilometre grid.

**Figure A1:**
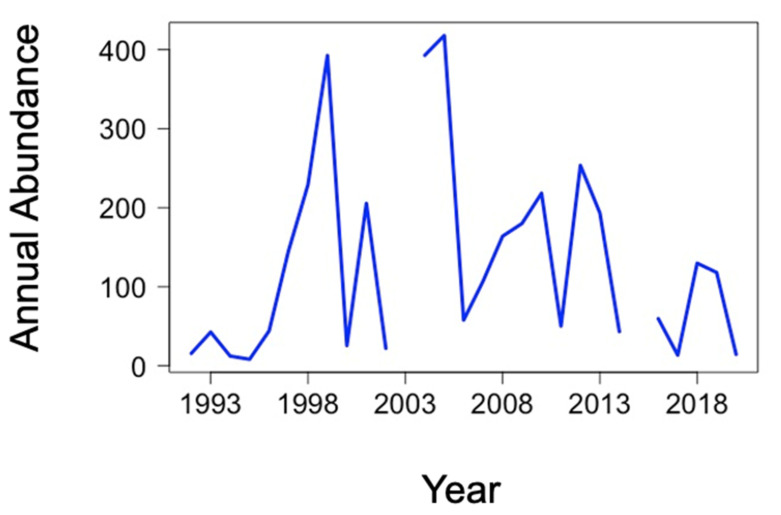
Annual abundance of *Calliphora vicina* in the eastern Kerguelen Islands over 27-year period (1993–2020).

Long-term monitoring of the distribution of *C. vicina* at the Kerguelen Islands revealed that the fly was found widely across the sampled points in the eastern part of the archipelago in earlier sampling years (roughly until 2012), while it was less commonly found from 2018 onwards (Figure 3 and Figure A1). Large-scale surveying conducted between 2004–2007 confirmed the expansion of *C. vicina* across the eastern part of the archipelago, including islands within the Golfe du Morbihan. Flying adult individuals have also been seen on Ile Howe in the northern part of the islands. In the more recent years (2017–2021), the species’ distribution has shifted more towards the interior of the Péninsule Courbet (Figure 3). Large variations in *C. vicina* abundance were observed across years (Figure A1). Total abundance per year peaked during the mid-1990s to the mid-2000s, with a large decrease (approx. 300 individuals) in the number of individuals caught per year between the peak and recent years (Figure A1).

### 3.4. Phenological Niche of Calliphora vicina

**Figure 4:**
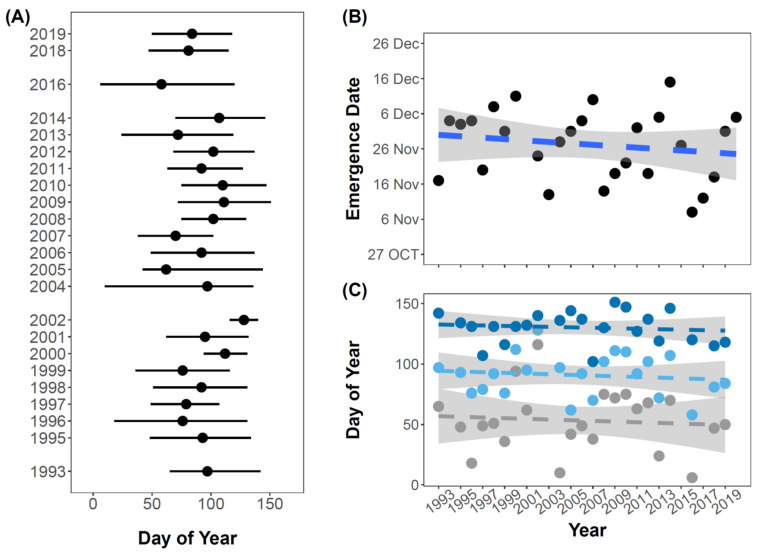
Estimated phenology of a population of *C. vicina* at the Kerguelen Islands from long-term monitoring data collected during 1993–2019. (**A**) Period of *C. vicina* activity in the years 1993–2019. The line indicates the duration of activity (time from onset to end of activity) and the points indicate the peak of *C. vicina* activity period. (**B**) Day of year where the required average accumulated degree days of 439 (calculated from 200th day of year–austral mid-winter when adult flies are inactive) for adult *C. vicina* emergence has been reached and (**C**) temporal shifts in emergence phenology of *C. vicina* from 1993–2019. The grey line and points (bottom) represent onset; the light blue line and points (middle) represent peak; and the dark blue line and points represent end of emergence (top). None of the trends were significant.

The seasonal activity period of *C. vicina* showed that emergence varied markedly among years (Figure 4A). In some years, the activity period began in early January, whereas in other years it did not begin until April. The peak varied between March and early May, and the activity period ended between April and late May. This corresponds to an active period stretching from the austral summer to autumn. Thus, adult *C. vicina* show no, or very little, activity during the winter where adults, or the other developmental stages, are likely in a state of quiescence. Since average daily temperatures remain below or around 2 °C during winter (Figure 2B), the fly development is likely slow or completely paused during this period.

Our detailed investigation of the degree days, calculated from hourly temperature data available from 1993 to 2020, showed that the day of year when sufficient accumulated degree days was reached for the adult emergence of *C. vicina* did not change significantly during the study period (1993–2020) (Figure 4B). The day of the year where enough degree days were accumulated varied by a little over a month (for example, adults emerged on the 15th of December in 2014 and on the 8th of November in 2016). Similarly, our analysis of a potential temporal change in the phenology of *C. vicina* also showed no significant change in the onset, peak, or end of *C. vicina*’s activity during the study period 1993–2020 (Figure 4C, onset: R^2^ = 0.0008, F_1,21_ = 0.15, *p* = 0.90; peak: R^2^ = 0.02, F_1,21_ = 0.43, *p* = 0.52; end: R^2^ = 0.01, F_1,21_ = 0.28, *p* = 0.60). The peak and end of emergence of *C. vicina* was strongly positively associated with air temperature across years (Table A1), i.e., both the peak and end of emergence advanced as the temperature increased. No significant effect of air temperature was found for the onset of emergence. Precipitation was a poor predictor for all three phenological responses (Table A1).

## 4. Discussion

A temporal lag between the introduction and proliferation is a common dynamic in biological invasions [44,45] and can be caused by a range of factors. In the context of climate change, changing environmental conditions, such as warming temperatures, can facilitate the proliferation and expansion of non-native ‘sleeper’ populations that otherwise would exist in small numbers or patches with suitable microclimates [4]. The isolation of the Kerguelen population of *C. vicina* in a harsh, but changing environment, has presented an interesting opportunity to examine the effects of climate change on the establishment and proliferation of this non-native fly. After its initial observation in 1978, *C. vicina* individuals are thought to have persisted within the heated buildings of the Port-aux-Français research station, and may have been frequently re-supplied with adults unintentionally transported by the station supply ships. During the 1970′s, temperatures increased rapidly (Figure 3), which may have improved the environmental conditions for *C. vicina* [16]. Our examination of accumulated degree days showed a marked change pre- and post-introduction, as thermal conditions became more than sufficient for the blowfly’s development during the following decade (1980s) and onwards. This study provides evidence that increasing the temperature in this period likely facilitated the establishment of this blowfly at the Kerguelen Islands, subsequently facilitating its spread throughout eastern parts of the islands, where conditions are typically milder.

After its establishment, the Kerguelen population of *C. vicina* may also have progressively developed thermal requirements better adapted to cool conditions. Unfortunately, we could not test this hypothesis directly by comparing thermal tolerances of individuals from the initial introduction phase because the thermal tolerance of flies introduced in the 1970s was never studied. However, our experimental results showed limited development at low temperatures (4 °C), while in other cool regions, individuals can emerge quickly at even cooler temperatures. For example, [46] showed that hatching could occur in as little as 13 days during incubation at 3.5 °C for cultures sampled from Northern England, while our samples took over 30 days to hatch at 4 °C. Additionally, the minimum developmental temperatures for this species can be as low as 1 °C, depending on the geographic region (for a summary of minimum developmental temperatures, see [47]). Additionally, the number of days to eclosion was similar in our 16 °C treatment to that of cultures reared at 15 °C originating from Northern Italy [26]. Although not conclusive, this suggests that the Kerguelen population of *C. vicina* may not yet have developed thermal adaptations to the cooler local conditions. Instead, it is more likely that this species is benefitting from a recent shift towards more favourable temperatures, as evidenced by our results.

The phenology results showed that the active period coincided with the austral summer (Figure 4A), which is a period of high biological activity locally. This overlap provides many opportunities for *C. vicina* to interact with other species in the community, although future studies of community-wide phenological trends are needed to better understand the temporal overlap with interacting species. In addition, *C. vicina* seems to be phenologically sensitive to a warming climate, as we found that the peak and end of its emergence showed significant advancements with increasing temperature (Table A1). This indicates that *C. vicina* benefits from current climatic changes, and should be able to persist if temperatures continue to rise at the Kerguelen Islands. As such, any future benefits from a warming climate could further intensify its effects on the local community. The phenology results also showed large variations in the timing of emergence and length of the activity period. Further, no significant temporal trends in emergence were found. These findings might be explained by the large interannual variation, and the absence of significant temporal change in temperature since the late 80′s. This large phenological variation suggests a high level of phenotypic plasticity to climatic variation in this species, as is often seen for many successful non-native species (e.g., [48]). This contributes to their ability to quickly adjust to new conditions [49], and allows this species to track climate shifts more closely than native species [20].

Climate change is predicted to increase the range and shorten the generation time of many invasive insects like *C. vicina*. For example, it may also have contributed to problematic outbreaks of the mountain pine beetle (*Dendroctonus ponderosae*, Hopkins 1902) in North America [50] and the processionary moth (*Thaumetopoea pityocampa*, Denis and Schiffermüller 1775) in Europe [51]. Phenological shifts, induced by a warming climate, may lead to extended growing and breeding seasons and consequently an increase in the number of generations per year, as projected for the spruce bark beetle (*Ips typographus*, Linnaeus 1758) in Sweden [52]. Such an increase in the number of generations has already been documented for the mountain pine beetle (*D. ponderosae*) in North America [53]. While we did not perform a detailed investigation of the number of generations produced by *C. vicina* in a season in this study, the phenology curves derived for some of the most recent years (2016, 2017 and 2020) show bimodal distributions in abundance across the breeding season (Figure S1). This may suggest that climate change is progressively enabling the flies to produce additional generations through a lengthening of the breeding season, as has been observed in a number of highly plastic multivoltine insects, such as lepidoptera [54,55].

Long-term monitoring revealed that the Kerguelen *C. vicina* population seems to exhibit recurrent boom-bust dynamics, with a period of around two and a half years (Figure A1). This dynamic can be driven by mechanisms specifically associated with non-native populations, general ecological factors, or a mix of multiple mechanisms [49]. However, the sampling is limited, and without experimental manipulation, we are unable to identify the underlying factors causing these dynamics. In summary, our findings highlight the crucial need for the continued monitoring of *C. vicina* on the Kerguelen Islands to ensure that we can track the responses of this species when breeding seasons are extended under continued climate warming. Moreover, while the long-term monitoring program at the Kerguelen Islands provides a unique opportunity to detect phenological and population trends for this successful invader in relation to a changing climate, the spatial variation in the response of *C. vicina* populations to climate change remains unknown. Future studies could consider the response of intraspecific differences in phenological responses to climate change by including the monitoring of different populations of *C. vicina* from different habitat types, as well as the monitoring of other life stages, which could respond differently to climate warming.

In the early 2000s, the future of *C. vicina* in the sub-Antarctic was less certain, as it was thought that several successive cold years could eliminate the local population through the inhibition of development (136-SUBANTECO, French Polar Institute). However, those expected successive cold years never came, and *C. vicina* persisted. With the ever-present human activity on the Kerguelen Islands, and thus the persistent need for goods transported with supply vessels, *C. vicina* is likely to be rapidly reintroduced, even in the case of a complete extirpation event. This, combined with continually increasing average temperatures and the species’ rapid response to warming, means that it is likely ‘here to stay’ at the Kerguelen Islands. Thus, the significance of this species as an invader at the Kerguelen Islands must be better understood, so its impacts can be managed.

Although the ecological impacts of the *C. vicina* invasion have only rarely been considered, we have identified at least three main routes through which *C. vicina* could alter ecological dynamics in the Kerguelen Islands. These are through *C. vicina*’s (i) use of carrion resources, (ii) its potential role as a prey item for other species, and (iii) its potential role as a pollinator, or, owing to its good flying capacity, as a transporter of plant propagules that could help alien plants to establish and expand their range. The first primary pathway, the role of *C. vicina* as a carrion consumer, could impact the local ecosystem in a number of ways. For example, *C. vicina* larvae may compete with other carrion consumers, such as the endemic native wingless fly *Anatalanta aptera* (Eaton 1875), as seen in other native and non-native carrion blowfly pairings [29]. However, experimental work by Chevrier, Vernon, and Frenot [27] showed that direct competition between these species does not occur when resources are abundant. However, indirect competition may occur, as some blowflies can stagger their arrival time or entirely avoid occupied carrion to reduce direct competition and intraguild predation [56,57]. Relative to *A. aptera*, *C. vicina*’s fast development and superior dispersal ability could allow it to colonise and exploit carrion resources more easily and potentially exclude *A. aptera*, especially in areas with low carrion availability [16]. This, combined with threats from other non-native species, such as the predatory beetle *Merizodus soledadinus* (Guérin-Méneville 1830), could threaten the long-term survival of *A. aptera* [16,58].

The horizontal transmission of pathogens through shared material, such as carrion, is also possible [59] and could lead to spillover/spillback dynamics [60,61,62] with pathogens or parasites originating in *C. vicina*, other decomposers, or both. Spillover is when a pathogen or parasite ‘spills’ from one population to another, while spillback occurs when a newly infected population becomes a pathogen source for the original reserve population [8]. Other blowflies have also been shown to spread pathogens, such as anthrax (Bacillus anthracis) from carcasses to the surrounding environment and vegetation [60], and it is possible that *C. vicina* could also affect pathogen dynamics locally. Additionally, the presence of carrion-feeding *C. vicina* larvae could affect the larger ecosystem through its effects on the carrion microhabitat and the rate of nutrient cycling. Blowfly larvae can create a ‘larval mass effect’, where aggregated larvae raise the temperature in the carrion microhabitat, which may promote their survival in two ways. One is that higher temperatures promote rapid growth, thereby reducing the time spent in a vulnerable developmental stage [63], especially in environments with relatively low temperatures. The other is that higher temperatures may confer a competitive advantage of *C. vicina* relative to other species [64]. Higher microhabitat temperatures could improve conditions for micro decomposers and accelerate decomposition, thus altering the nutrient cycle.

The second major way that the *C. vicina* invasion could affect the local community is by acting as a novel prey item for predators, such as the invasive house mouse (*Mus musculus*) and the predatory carabid beetle *M. soledadinus*, especially in its larval stage. Once *C. vicina* reaches the adult stage, it is unlikely to be a significant prey item for any local species because there are no predators adapted to aerial insect prey in the community (as there are no native flying insects at the Kerguelen Islands). *C. vicina* could also act as prey for future non-native species and facilitate their establishment, proliferation, and expansion regionally. Finally, in addition to potentially altering decomposition dynamics and acting as a prey species, *C. vicina* could affect the local ecosystem by facilitating the future invasion of pollination-dependent plants [65]. Many sub-Antarctic islands lack pollinating insects, and most native plants are pollinated by wind. A high abundance of *C. vicina*, which is anthophilous and consumes nectar in the adult stage, represents an opportunity for insect-pollinated plants to establish, spread, and proliferate in future sub-Antarctic communities [32,65]. Through its potential interactions with other species as prey or pollinator, *C. vicina* could facilitate the invasion of diverse species and contribute to an ‘invasional meltdown’ in the Kerguelen Islands [8,66].

## 5. Conclusions

By combining data from multiple sources (long-term ecological monitoring, experimental biological, and meteorological), we identified a link between warming temperatures and both the long-term establishment and seasonal behaviour of *C. vicina* in the Kerguelen Islands. We have shown that this species is sensitive to climate change, and will likely continue to benefit from rising temperatures, possibly to the point of increasing the number of generations produced each year. Polar ecosystems were left relatively ‘untouched’ until recent times and are now facing intensifying pressure from the interacting global disturbances of climate change and biological invasions. As with other invaders in these ecosystems, *C. vicina* was introduced to the Kerguelen ecosystem and was able to persist there long enough for it to benefit from warming conditions. Due to the high potential for ecological effects, and the sensitive and unique features of the Kerguelen Islands, we emphasise the necessity of continued monitoring of this population and studies of its significance in the terrestrial sub-Antarctic ecological network. As total eradication (e.g., by insecticide use) is not possible within this ecosystem due to risks presented to non-target invertebrates, the effects of this species must be identified to prioritise efforts for strategic and targeted management. This can reduce any negative impacts to at-risk species and communities and help determine whether action must be taken to limit its spread across the islands. It is imperative to prioritise a better understanding of the invasion process of species introduced to cold regions, and how their fates interact with a rapidly changing climate, because as humanity’s technological advances facilitate our own ability to exploit polar regions, they also provide more opportunities for biological invaders to do the same.

## Figures and Tables

**Table 1:** Total number of days to reach each developmental stage of *Calliphora vicina*‘s life cycle from hatching ^a^, and number of degree days (DD) to reach adulthood ^b^, in the Kerguelen Islands strain reared under different thermal conditions (4, 8, 12, 16, and 20 °C and ambient temperatures (ambient conditions, AC)) at Port-Aux-Français, Kerguelen Islands. Ambient temperatures were measured at 2 m height by a weather station at Port-Aux-Français. For the calculations, 2 °C was selected as the minimum developmental temperature threshold.

	Days to Stage						
Temp. (°C)	Hatch	1st Larval Instar	2nd Larval Instar	3rd Larval Instar	Pupa	Adult	Total DD
4	31	-	-	-	-	-	-
8	4.5	6	9	18.5	23	92.8	556.8
12	2	4	5	7.5	12	41.5	415
16	1	3	3	5	7	29.9	418.6
20	1	2	2.5	4	5	22.6	406.8
AC	4	3	4.5	10	16	73	397.8

^a^ For each developmental stage, data presented in the table indicate the median time elapsed (in days) since eggs were laid. The only exception is for ‘Hatch’, which reports the time it took for observing the first 10 percent hatch. Data were obtained from a minimum of three independent rearing experiments for each temperature condition. ^b^ Total degree days were calculated by considering the median time required to reach adulthood from a developmental temperature threshold of 2 °C. Under constant temperature, the number of degree days are the sum across days of temperature above the developmental threshold × day(s)—for instance, at 8 °C, it took 92.8 days to reach adulthood, and thus the total degree days were (8 °C–2 °C) × 92.8 = 556.8. The data also makes it possible to calculate the necessary degree days in between two consecutive developmental stages. By keeping the example of the 8 °C rearing condition, it took 9.5 (18.5–9) days to pass from the 2nd to the 3rd larval instar, thus meaning 57 degree days (9.5 × 6).

## Data Availability

The data presented in this study are openly available in FigShare at 10.6084/m9.figshare.21744299, accessed on 17 December 2022. R scripts from the analyses in this article are available in GitHub at https://github.com/davidrenault/Calliphora-data-and-script, accessed on 17 December 2022.

## Supplementary Materials

The following supporting information can be downloaded at: https://www.mdpi.com/article/10.3390/biology12010111/s1, Figure S1: Seasonal phenological curves calculated from the long-term monitoring data of *Calliphora vicina* for the years 1993–2020 from which the phenological events; Onset, peak and end of activity have been estimated from.

## Appendix A

This appendix includes one supporting table and figure.

**Table A1:** Linear regression models of the onset, peak, and end of emergence of *Calliphora vicina* in response to climate variables: average air temperature (temp) and precipitation (precip) 30 days prior to each phenological response variable, as well as a model including both variables (temperature + precipitations).

	Emergence Onset				Emergence Peak				Emergence End			
Model	Coef.	SE	R^2^	*p*	Coef.	SE	R^2^	*p*	Coef.	SE	R^2^	*p*
Temp.	0.13	5.78	2.54 × 10^−5^	0.98	−11.00	2.00	0.57	<0.0001	−9.54	1.81	0.55	<0.0001
Precip.	−1.77	6.90	0.003	0.80	3.77	4.52	0.03	0.41	−2.65	2.20	0.02	0.24
Temp. + Precip.	−1.45	8.11	0.005	0.86	1.60	2.43	0.54	0.52	−2.74	2.00	0.57	0.19

## References

[1] Reid A.J., Carlson A.K., Creed I.F., Eliason E.J., Gell P.A., Johnson P.T.J., Kidd K.A., MacCormack T.J., Olden J.D., Ormerod S.J. (2019). Emerging Threats and Persistent Conservation Challenges for Freshwater Biodiversity. Biol. Rev..

[2] Pyšek P., Hulme P.E., Simberloff D., Bacher S., Blackburn T.M., Carlton J.T., Dawson W., Essl F., Foxcroft L.C., Genovesi P. (2020). Scientists’ Warning on Invasive Alien Species. Biol. Rev..

[3] Dukes J.S., Mooney H.A. (1999). Does Global Change Increase the Success of Biological Invaders?. Trends Ecol. Evol..

[4] Hulme P.E. (2017). Climate Change and Biological Invasions: Evidence, Expectations, and Response Options. Biol. Rev. Camb. Philos. Soc..

[5] Vilà M., Hulme P.E., Vilà M., Hulme P.E. (2017). Non-Native Species, Ecosystem Services, and Human Well-Being. Impact of Biological Invasions on Ecosystem Services.

[6] Flood P.J., Duran A., Barton M., Mercado-Molina A.E., Trexler J.C. (2020). Invasion Impacts on Functions and Services of Aquatic Ecosystems. Hydrobiologia.

[7] Frost C.M., Allen W.J., Courchamp F., Jeschke J.M., Saul W.-C., Wardle D.A. (2019). Using Network Theory to Understand and Predict Biological Invasions. Trends Ecol. Evol..

[8] Daly E.Z., Chabrerie O., Massol F., Facon B., Hess M.C.M., Tasiemski A., Grandjean F., Chauvat M., Viard F., Forey E. (2023). A Synthesis of Biological Invasion Hypotheses Associated with the Introduction-Naturalisation-Invasion Continuum. Oikos.

[9] Mahoney P.J., Beard K.H., Durso A.M., Tallian A.G., Long A.L., Kindermann R.J., Nolan N.E., Kinka D., Mohn H.E. (2015). Introduction Effort, Climate Matching and Species Traits as Predictors of Global Establishment Success in Non-Native Reptiles. Divers. Distrib..

[10] Feng Y.-L., Du D., van Kleunen M. (2022). Global Change and Biological Invasions. J. Plant Ecol..

[11] Porter E.M., Bowman W.D., Clark C.M., Compton J.E., Pardo L.H., Soong J.L. (2013). Interactive Effects of Anthropogenic Nitrogen Enrichment and Climate Change on Terrestrial and Aquatic Biodiversity. Biogeochemistry.

[12] Essl F., Lenzner B., Bacher S., Bailey S., Capinha C., Daehler C., Dullinger S., Genovesi P., Hui C., Hulme P.E. (2020). Drivers of Future Alien Species Impacts: An Expert-Based Assessment. Glob. Chang. Biol..

[13] Gvoždík L. (2018). Just What Is the Thermal Niche?. Oikos.

[14] Renault D., Leclerc C., Colleu M.-A., Boutet A., Hotte H., Colinet H., Chown S.L., Convey P. (2022). The Rising Threat of Climate Change for Arthropods from Earth’s Cold Regions: Taxonomic Rather than Native Status Drives Species Sensitivity. Glob. Chang. Biol..

[15] Stevens G.C. (1989). The Latitudinal Gradient in Geographical Range: How so Many Species Coexist in the Tropics. Am. Nat..

[16] Lebouvier M., Laparie M., Hullé M., Marais A., Cozic Y., Lalouette L., Vernon P., Candresse T., Frenot Y., Renault D. (2011). The Significance of the Sub-Antarctic Kerguelen Islands for the Assessment of the Vulnerability of Native Communities to Climate Change, Alien Insect Invasions and Plant Viruses. Biol. Invasions.

[17] Janion-Scheepers C., Phillips L., Sgrò C.M., Duffy G.A., Hallas R., Chown S.L. (2018). Basal Resistance Enhances Warming Tolerance of Alien over Indigenous Species across Latitude. Proc. Natl. Acad. Sci. USA.

[18] Menzel A., Sparks T.H., Estrella N., Koch E., Aasa A., Ahas R., Alm-Kübler K., Bissolli P., Braslavská O., Briede A. (2006). European Phenological Response to Climate Change Matches the Warming Pattern. Glob. Chang. Biol..

[19] Parmesan C., Yohe G. (2003). A Globally Coherent Fingerprint of Climate Change Impacts across Natural Systems. Nature.

[20] Wolkovich E.M., Cleland E.E. (2011). The Phenology of Plant Invasions: A Community Ecology Perspective. Front. Ecol. Environ..

[21] Wolkovich E.M., Cleland E.E. (2014). Phenological Niches and the Future of Invaded Ecosystems with Climate Change. AoB PLANTS.

[22] Arias P., Bellouin N., Coppola E., Jones R., Krinner G., Marotzke J., Naik V., Palmer M., Plattner G.-K., Rogelj J., Masson-Delmotte V., Zhai P., Pirani A., Conners S.L., Péan C., Berger S., Caud N., Chen Y., Goldfarb L. (2021). Climate Change 2021: The Physical Science Basis. Contribution of Working Group I to the Sixth Assessment Report of the Intergovernmental Panel on Climate Change.

[23] Pauchard A., Milbau A., Albihn A., Alexander J., Burgess T., Daehler C., Englund G., Essl F., Evengård B., Greenwood G.B. (2016). Non-Native and Native Organisms Moving into High Elevation and High Latitude Ecosystems in an Era of Climate Change: New Challenges for Ecology and Conservation. Biol. Invasions.

[24] Bauer A., Bauer A.M., Tomberlin J.K. (2020). Impact of Diet Moisture on the Development of the Forensically Important Blow Fly *Cochliomyia macellaria* (Fabricius) (Diptera: Calliphoridae). Forensic Sci. Int..

[25] Marchenko M.I. (2001). Medicolegal Relevance of Cadaver Entomofauna for the Determination of the Time of Death. Forensic Sci. Int..

[26] Defilippo F., Bonilauri P., Dottori M. (2013). Effect of Temperature on Six Different Developmental Landmarks within the Pupal Stage of the Forensically Important Blowfly *Calliphora vicina* (Robineau-Desvoidy) (Diptera: Calliphoridae). J. Forensic Sci..

[27] Chevrier M., Vernon P., Frenot Y. (1997). Potential Effects of Two Alien Insects on a Sub-Antarctic Wingless Fly in the Kerguelen Islands. Antarctic Communities: Species, Structure and Survival.

[28] Wells J.D., Greenberg B. (1992). Interaction between *Chrysomya rufifacies* and *Cochliomyia macellaria* (Diptera: Calliphoridae): The Possible Consequences of an Invasion. Bull. Entomol. Res..

[29] Carmo R.F.R., Vasconcelos S.D., Brundage A.L., Tomberlin J.K. (2018). How Do Invasive Species Affect Native Species? Experimental Evidence from a Carrion Blowfly (Diptera: Calliphoridae) System. Ecol. Entomol..

[30] Frenot Y., Chown S.L., Whinam J., Selkirk P.M., Convey P., Skotnicki M., Bergstrom D.M. (2005). Biological Invasions in the Antarctic: Extent, Impacts and Implications. Biol. Rev. Camb. Philos. Soc..

[31] Bellard C., Rysman J.-F., Leroy B., Claud C., Mace G.M. (2017). A Global Picture of Biological Invasion Threat on Islands. Nat. Ecol. Evol..

[32] Hullé M., Vernon P. (2021). Terrestrial Macro-Arthropods of the Sub-Antarctic Islands of Possession (Crozet Archipelago) and Kerguelen: Inventory of Native and Non-Native Species. Zoosystema.

[33] Krikken J., Huijbregts J. (2001). Insects as Forensic Informants: The Dutch Experience and Procedure. Proc. Exp. Appl. Entomol..

[34] Vinogradova E.B., Zinovjeva K.B. (1972). Maternal Induction of Larval Diapause in the Blowfly, *Calliphora vicina*. J. Insect Physiol..

[35] Graham-Smith G.S. (1916). Observations on the Habits and Parasites of Common Flies. Parasitology.

[36] Vinogradova E.B., Taylor F., Karban R. (1986). Geographical Variation and Ecological Control of Diapause in Flies. The Evolution of Insect Life Cycles.

[37] Aak A., Birkemoe T., Leinaas H.P. (2011). Phenology and Life History of the Blowfly *Calliphora vicina* in Stockfish Production Areas. Entomol. Exp. Appl..

[38] Allen J.C. (1976). A Modified Sine Wave Method for Calculating Degree Days 1. Environ. Entomol..

[39] Donovan S.E., Hall M.J.R., Turner B.D., Moncrieff C.B. (2006). Larval Growth Rates of the Blowfly, *Calliphora vicina*, over a Range of Temperatures. Med. Vet. Entomol..

[40] Putman R.J. (1977). Dynamics of the Blowfly, *Calliphora erythrocephala*, Within Carrion. J. Anim. Ecol..

[41] Simpson G.L. (2018). Modelling Palaeoecological Time Series Using Generalised Additive Models. Front. Ecol. Evol..

[42] Welcome to the QGIS Project!. https://qgis.org/en/site/.

[43] Wood S.N. (2017). An Introduction with R, Second Edition.

[44] Rouget M., Robertson M.P., Wilson J.R.U., Hui C., Essl F., Renteria J.L., Richardson D.M. (2016). Invasion Debt—Quantifying Future Biological Invasions. Divers. Distrib..

[45] Coutts S.R., Helmstedt K.J., Bennett J.R. (2018). Invasion Lags: The Stories We Tell Ourselves and Our Inability to Infer Process from Pattern. Divers. Distrib..

[46] Davies L., Ratcliffe G.G. (1994). Development Rates of Some Pre-Adult Stages in Blowflies with Reference to Low Temperatures. Med. Vet. Entomol..

[47] Salimi M., Rassi Y., Oshaghi M., Chatrabgoun O., Limoee M., Rafizadeh S. (2018). Temperature Requirements for the Growth of Immature Stages of Blowflies Species, *Chrysomya albiceps* and *Calliphora vicina*, (Diptera:Calliphoridae) under Laboratory Conditions. Egypt. J. Forensic Sci..

[48] Chown S.L., Marais E., Terblanche J.S., Klok C.J., Lighton J.R.B., Blackburn T.M. (2007). Scaling of Insect Metabolic Rate Is Inconsistent with the Nutrient Supply Network Model. Funct. Ecol..

[49] Strayer D.L., D’Antonio C.M., Essl F., Fowler M.S., Geist J., Hilt S., Jarić I., Jöhnk K., Jones C.G., Lambin X. (2017). Boom-Bust Dynamics in Biological Invasions: Towards an Improved Application of the Concept. Ecol. Lett..

[50] Raffa K.F., Aukema B.H., Bentz B.J., Carroll A.L., Hicke J.A., Turner M.G., Romme W.H. (2008). Cross-Scale Drivers of Natural Disturbances Prone to Anthropogenic Amplification: The Dynamics of Bark Beetle Eruptions. BioScience.

[51] Battisti A., Stastny M., Netherer S., Robinet C., Schopf A., Roques A., Larsson S. (2005). Expansion of Geographic Range in the Pine Processionary Moth Caused by Increased Winter Temperatures. Ecol. Appl..

[52] Jönsson A.M., Appelberg G., Harding S., Bärring L. (2009). Spatio-Temporal Impact of Climate Change on the Activity and Voltinism of the Spruce Bark Beetle, *Ips typographus*. Glob. Chang. Biol..

[53] Mitton J.B., Ferrenberg S.M., Benkman N.H.E.C.W. (2012). Mountain Pine Beetle Develops an Unprecedented Summer Generation in Response to Climate Warming. Am. Nat..

[54] Pöyry J., Leinonen R., Söderman G., Nieminen M., Heikkinen R.K., Carter T.R. (2011). Climate-Induced Increase of Moth Multivoltinism in Boreal Regions. Glob. Ecol. Biogeogr..

[55] Macgregor C.J., Thomas C.D., Roy D.B., Beaumont M.A., Bell J.R., Brereton T., Bridle J.R., Dytham C., Fox R., Gotthard K. (2019). Climate-Induced Phenology Shifts Linked to Range Expansions in Species with Multiple Reproductive Cycles per Year. Nat. Commun..

[56] Rosati J.Y. (2014). Spatial and Temporal Variability in the Carrion Insect Community: Using Blow Flies (Family: Calliphoridae) as a Model System to Study Coexistence Mechanisms at Multiple Scales. Doctoral Thesis.

[57] Galindo L.A., Moral R.A., Moretti T.C., Godoy W.A.C., Demétrio C.G.B. (2016). Intraguild Predation Influences Oviposition Behavior of Blow Flies (Diptera: Calliphoridae). Parasitol. Res..

[58] Lebouvier M., Lambret P., Garnier A., Convey P., Frenot Y., Vernon P., Renault D. (2020). Spotlight on the Invasion of a Carabid Beetle on an Oceanic Island over a 105-Year Period. Sci. Rep..

[59] Vicente J., VerCauteren K., Olea P.P., Mateo-Tomás P., Sánchez-Zapata J.A. (2019). The Role of Scavenging in Disease Dynamics. Carrion Ecology and Management.

[60] Basson L., Hassim A., Dekker A., Gilbert A., Beyer W., Rossouw J., van Heerden H. (2018). Blowflies as Vectors of *Bacillus anthracis* in the Kruger National Park. Koedoe.

[61] Daszak P., Cunningham A.A., Hyatt A.D. (2000). Emerging Infectious Diseases of Wildlife—Threats to Biodiversity and Human Health. Science.

[62] Eppinga M.B., Rietkerk M., Dekker S.C., De Ruiter P.C., Van der Putten W.H., Van der Putten W.H. (2006). Accumulation of Local Pathogens: A New Hypothesis to Explain Exotic Plant Invasions. Oikos.

[63] Catts E.P. (1992). Problems in Estimating the Postmortem Interval in Death Investigations. J. Agric. Entomol..

[64] Richards C.S., Price B.W., Villet M.H. (2009). Thermal Ecophysiology of Seven Carrion-Feeding Blowflies in Southern Africa. Entomol. Exp. Appl..

[65] Convey P., Key R.S., Key R.J.D. (2010). The Establishment of a New Ecological Guild of Pollinating Insects on Sub-Antarctic South Georgia. Antarct. Sci..

[66] Simberloff D., Von Holle B. (1999). Positive Interactions of Nonindigenous Species: Invasional Meltdown?. Biol. Invasions.

