# Supplementary material for: Climate Change Helps Polar Invasives Establish and Flourish: Evidence from Long-Term Monitoring of the Blowfly Calliphora vicina
Source: Biology (Basel). 2023 Jan 10;12(1):111. doi: 10.3390/biology12010111 (PMC9856047; doi:10.3390/biology12010111)
Supplement: Supplementary file 1 [file biology-12-00111-s001.zip › biology-2136579-supplementary.pdf]

**Figure S1 -** Phenological curves derived for a population of *C. vicina* based on monitoring data (Abundance per Trap Day) across a season in the years 1993 – 2020. The vertical line in each plot indicates the calendar year change.

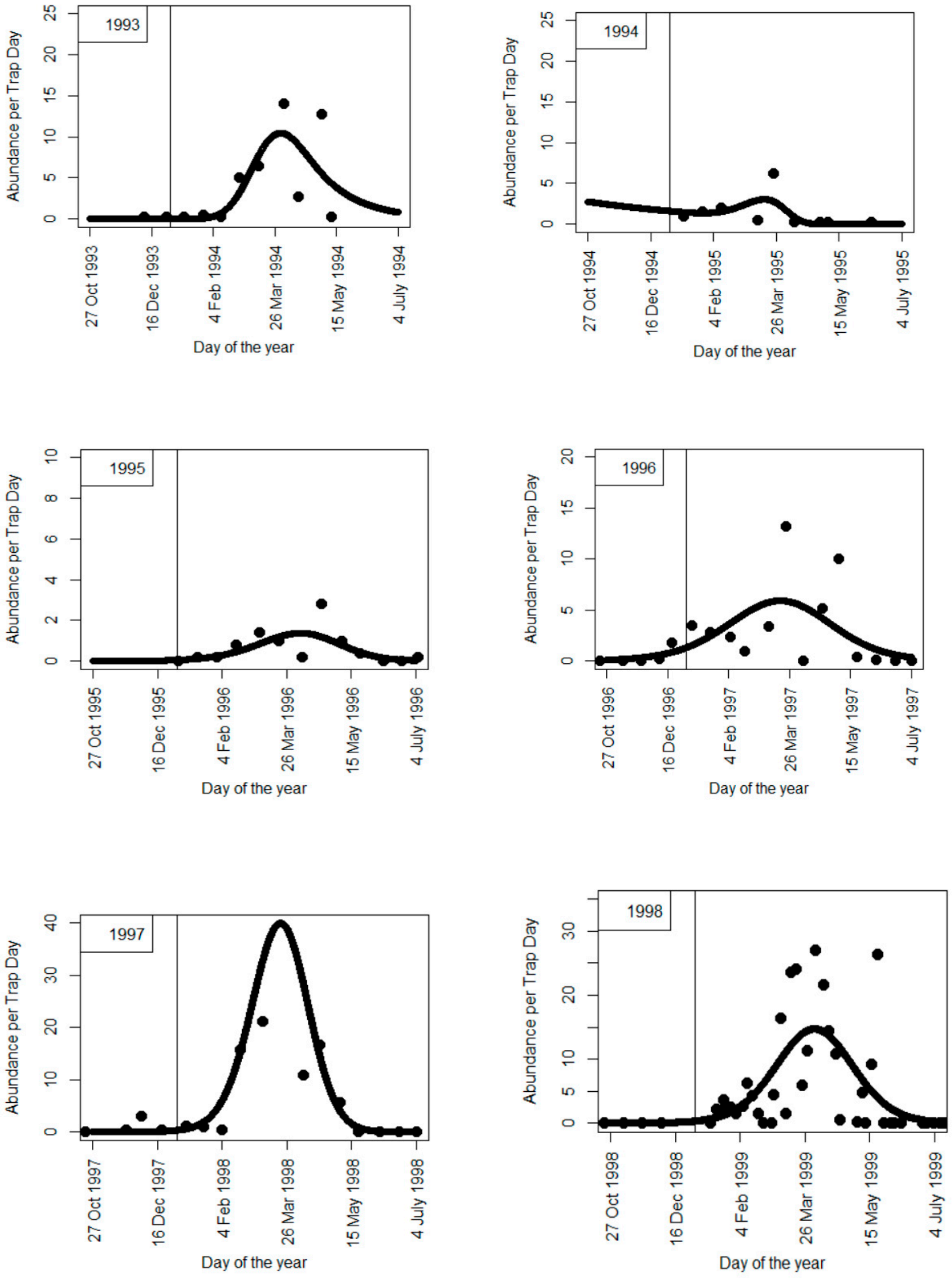

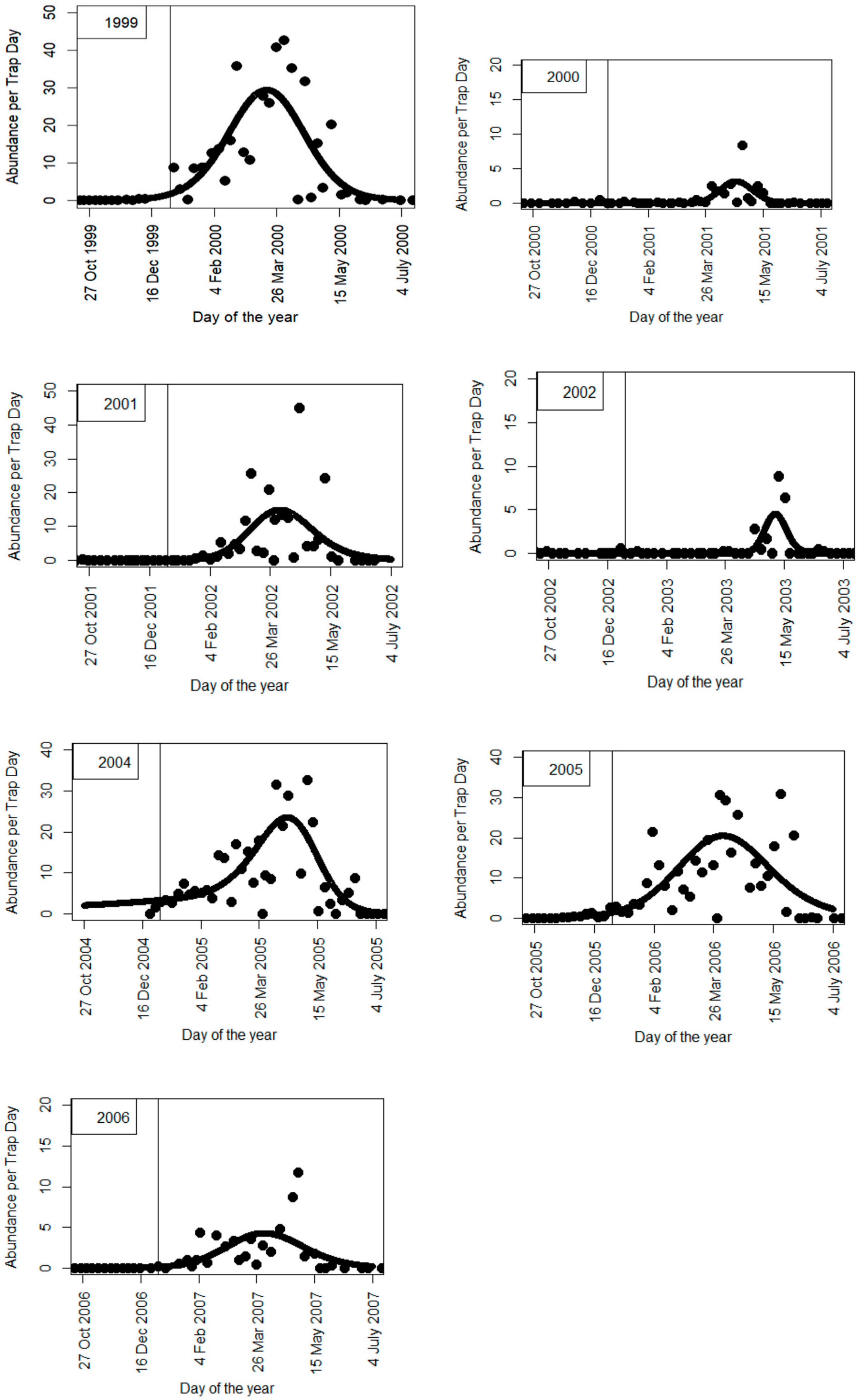
1999
Abundance per Trap Day
0
10
20
30
40
50
27 Oct 1999
16 Dec 1999
4 Feb 2000
26 Mar 2000
15 May 2000
4 July 2000
Day of the year
2000
Abundance per Trap Day
0
5
10
15
20
27 Oct 2000
16 Dec 2000
4 Feb 2001
26 Mar 2001
15 May 2001
4 July 2001
Day of the year
2001
Abundance per Trap Day
0
10
20
30
40
50
27 Oct 2001
16 Dec 2001
4 Feb 2002
26 Mar 2002
15 May 2002
4 July 2002
Day of the year
2002
Abundance per Trap Day
0
5
10
15
20
27 Oct 2002
16 Dec 2002
4 Feb 2003
26 Mar 2003
15 May 2003
4 July 2003
Day of the year
2004
Abundance per Trap Day
0
10
20
30
40
27 Oct 2004
16 Dec 2004
4 Feb 2005
26 Mar 2005
15 May 2005
4 July 2005
Day of the year
2005
Abundance per Trap Day
0
10
20
30
40
27 Oct 2005
16 Dec 2005
4 Feb 2006
26 Mar 2006
15 May 2006
4 July 2006
Day of the year
2006
Abundance per Trap Day
0
5
10
15
20
27 Oct 2006
16 Dec 2006
4 Feb 2007
26 Mar 2007
15 May 2007
4 July 2007
Day of the year

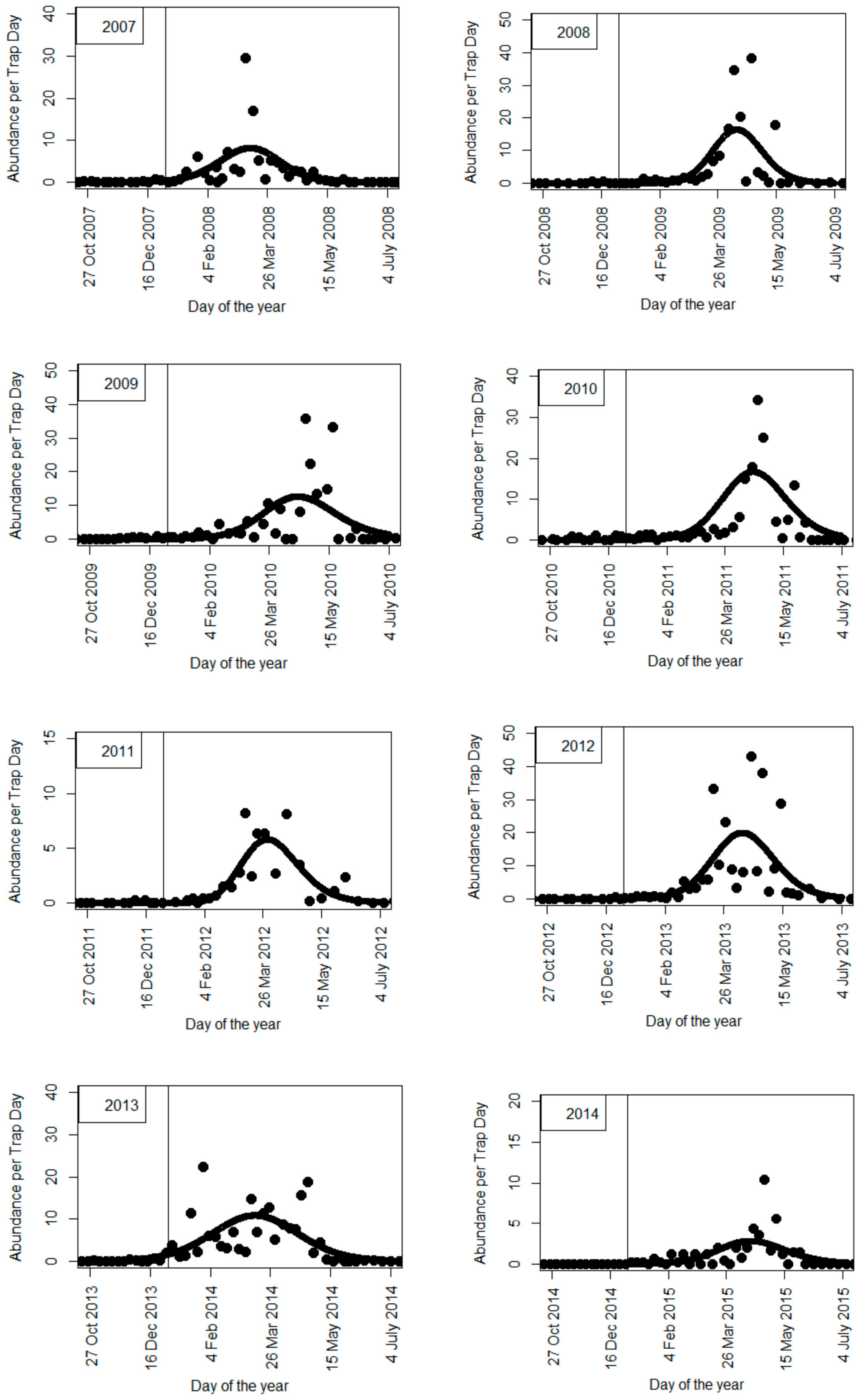
2007
Abundance per Trap Day
27 Oct 2007
16 Dec 2007
4 Feb 2008
26 Mar 2008
15 May 2008
4 July 2008
Day of the year
2008
Abundance per Trap Day
27 Oct 2008
16 Dec 2008
4 Feb 2009
26 Mar 2009
15 May 2009
4 July 2009
Day of the year
2009
Abundance per Trap Day
27 Oct 2009
16 Dec 2009
4 Feb 2010
26 Mar 2010
15 May 2010
4 July 2010
Day of the year
2010
Abundance per Trap Day
27 Oct 2010
16 Dec 2010
4 Feb 2011
26 Mar 2011
15 May 2011
4 July 2011
Day of the year
2011
Abundance per Trap Day
27 Oct 2011
16 Dec 2011
4 Feb 2012
26 Mar 2012
15 May 2012
4 July 2012
Day of the year
2012
Abundance per Trap Day
27 Oct 2012
16 Dec 2012
4 Feb 2013
26 Mar 2013
15 May 2013
4 July 2013
Day of the year
2013
Abundance per Trap Day
27 Oct 2013
16 Dec 2013
4 Feb 2014
26 Mar 2014
15 May 2014
4 July 2014
Day of the year
2014
Abundance per Trap Day
27 Oct 2014
16 Dec 2014
4 Feb 2015
26 Mar 2015
15 May 2015
4 July 2015
Day of the year

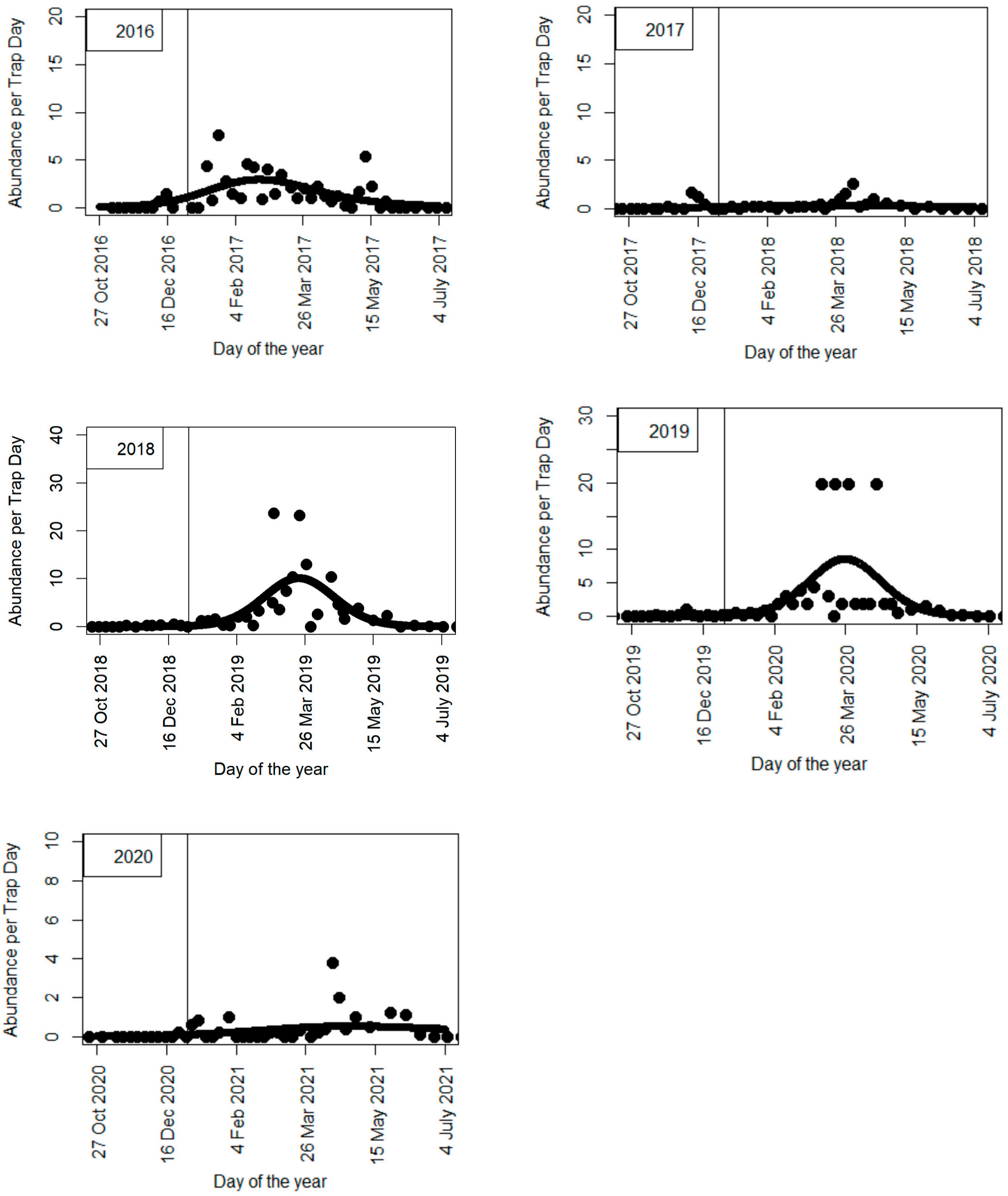
2016
2017
2018
2019
2020
Abundance per Trap Day
Day of the year
27 Oct 2016
16 Dec 2016
4 Feb 2017
26 Mar 2017
15 May 2017
4 July 2017
27 Oct 2017
16 Dec 2017
4 Feb 2018
26 Mar 2018
15 May 2018
4 July 2018
27 Oct 2018
16 Dec 2018
4 Feb 2019
26 Mar 2019
15 May 2019
4 July 2019
27 Oct 2019
16 Dec 2019
4 Feb 2020
26 Mar 2020
15 May 2020
4 July 2020
27 Oct 2020
16 Dec 2020
4 Feb 2021
26 Mar 2021
15 May 2021
4 July 2021
